# Progressive subcortical involvement as spinocerebellar ataxia type 3 advances

**DOI:** 10.1186/s13023-025-03803-3

**Published:** 2025-06-04

**Authors:** Pubing Yuan, Yonghua Huang, Minghui Dai, Xin Jin, Dingxin Zheng, Die Xiao, Lihua Deng, Peiling Ou, Linfeng Shi, Yifan Chen, Jian Wang, Wei Chen, Yuanchao Zhang, Chen Liu

**Affiliations:** 1 The Clinical Hospital of Chengdu Brain Science Institute, MOE Key Lab for Neuroinformation, University of Electronic Science and Technology of China, Chengdu, People’s Republic of China; 2 School of Life Science and Technology, University of Electronic Science and Technology of China, Chengdu, 610054 People’s Republic of China; 3 7T Magnetic Resonance Imaging Translational Medical Center, Southwest Hospital, Army Medical University (Third Military Medical University), Chongqing, People’s Republic of China; 4 Department of Radiology, Southwest Hospital, Army Medical University (Third Military Medical University), Chongqing, People’s Republic of China; 5 Department of Radiology, The 940 Hospital of Joint Logistics Support force of Chinese People’s Liberation Army, Lanzhou, People’s Republic of China; 6 MR Research Collaboration Team, Siemens Healthineers Ltd., Guangzhou, People’s Republic of China; 7 College of Health Solutions, Arizona State University, Tempe, AZ USA

**Keywords:** FSL-FIRST, *ATXN3*, Spinocerebellar ataxia type 3 (SCA3), Tract-based spatial statistics (TBSS)

## Abstract

**Background and objectives:**

Spinocerebellar ataxia type 3 (SCA3) is a progressive neurodegenerative disease characterized by heterogeneous motor and nonmotor manifestations. The progressive pattern of subcortical shape abnormalities and their associations with the clinical phenotypes in SCA3 remain unknown.

**Methods:**

Tract-based spatial statistics (TBSS) and FSL-FIRST were used to characterize the progressive patterns of the abnormalities in white matter microstructure and subcortical shape in four subgroups of SCA3 patients stratified based on disease duration (n = 56). These were compared to matched healthy control groups (n = 59).

**Results:**

TBSS analyses revealed a clear progressive pattern of white matter microstructural abnormalities throughout the course of SCA3, as indicated by an expanding topographic distribution of fractional anisotropy (FA) reductions that originated from the cerebellar peduncle. Vertex-based shape analyses uncovered an increasing number of affected subcortical structures in symptomatic patients as the disease progressed with concurrent inward atrophy and outward inflation in subcortical structures including the bilateral thalamus, caudate, putamen, pallidum, hippocampus and brainstem. Moreover, the localized shape changes of subcortical structures correlated bidirectionally with clinical measurements including the length of CAG repeats within the *ATXN3* gene, the scores on the scale of the assessment, the rating of ataxia, the instrumental activities of daily living scale, and the mini-mental state examination.

**Conclusion:**

We demonstrated progressive, localized, and bidirectional changes in the shape of subcortical structures that related to diverse clinical manifestations in SCA3, highlighting the pivotal role of localized shape abnormalities in contributing to the clinical heterogeneity of this disorder.

**Trial registration:**

Imaging genetics study the relationship between MJD1 gene and cognitive impairment with Spinocerebellar Ataxia type 3, ChiCTR1800019901. Registered 8 December 2018 and ChiCTR2000039434. Registered 28 October 2020, http://chictr.org.cn.

**Supplementary Information:**

The online version contains supplementary material available at 10.1186/s13023-025-03803-3.

## Introduction

Spinocerebellar ataxia type 3 (SCA3) is a progressive neurodegenerative disease caused by an abnormal CAG repeat expansion within the *ATXN3* gene, which encodes the ataxin-3 protein [1]. The expanded polyglutamine repeat in the ataxin-3 leads to neurotoxic aggregates that promote neuronal cell death [2]. Typical clinical symptoms of SCA3 include gait and limb ataxia, dysarthria, dysphagia, dystonia, bradykinesia, intentional tremor, and oculomotor disorders [3]. In addition to the motor impairments, patients with SCA3 often exhibit a varying degree of non-motor symptoms, such as abnormal cognitive and affective functions, depressive symptoms, and disinhibited behavior, among others [4]. However, the neuroanatomical substrates underlying the clinical heterogeneity of this disorder remain unknown.

Neuroimaging studies of SCA3 have identified prominent gray and white matter abnormalities in asymptomatic carriers, mainly involving the cerebellum [5–9]. Changes in the cerebellum have thus been thought of as strong predictors of ataxic symptoms [10]. In symptomatic patients, progressive pathologic changes involve a number of subcortical structures, which further exacerbate the motor and non-motor symptoms in this disorder [11]. Nevertheless, previous studies of subcortical structures using whole-brain voxel-based morphometry (VBM) and region of interest (ROI) based volumetry are inconsistent [12]; some studies observed reduced gray matter volume in the basal ganglia (lentiform nucleus, caudate, and putamen), while others did not. Such inconsistency likely relates to a conservative threshold due to a correction for multiple comparisons across the whole brain, insensitivity of ROI-based volumetric measurements [13, 14], and sample heterogeneities among studies such as different disease durations. Furthermore, different subregions of a single subcortical structure may also display substantial functional heterogeneities [15]. A more localized characterization of subcortical morphologic abnormalities in subgroups stratified based on duration of illness thus may help resolve the inconsistency among studies and contribute to the development of novel therapeutic interventions targeting specific nuclei of subcortical structures in patients with SCA3.

In the present study, we sought to examine the progressive pattern of localized shape abnormalities in subcortical structures in SCA3. This pattern becomes apparent as white matter microstructural abnormalities expand from focal abnormalities in the cerebellum to involve broader brain regions. Specifically, tract-based spatial statistics (TBSS) was used to characterize the white matter microstructural abnormalities in four subgroups of SCA3 patients with varying disease duration (n = 56) as compared to matched healthy control groups (n = 59). FSL-FIRST, a tool used for automatic segmentation of subcortical structures, was used to examine the localized subcortical abnormalities in each subgroup compared to the corresponding control group. We hypothesized that patients with SCA3 would show characteristic shape changes in subcortical structures as white matter microstructural abnormalities progress, contributing to the progression of clinical symptoms of this disorder.

## Materials and methods

### Participants

The present study initially recruited a total of 56 patients diagnosed with SCA3, using peripheral blood samples for genetic testing of CAG expansion. However, three patients manifesting SCA3 had to be excluded due to missing MRI data. To establish a control group, 59 healthy participants matching the age and gender criteria were recruited. These participants underwent MRI scans and clinical evaluations at the neurogenetic clinic of Southwest Hospital, Chongqing, China over the course of several years. Stringent exclusion criteria were applied to ensure that all control participants had no previous or family history of neurological disease, as well as no history of cerebellar or motor disorders.

Ethical approval for the study was obtained from the local ethics committee (KY2020191), and written informed consent was obtained from all participants prior to their involvement. The study was registered with the Chinese Clinical Trial Registry (ChiCTR, http://chictr.org.cn) under the registration numbers ChiCTR1800019901 and ChiCTR2000039434.

### Clinical measures and definition of subgroups

Clinical evaluations were performed on the same day as MRI scans. Specifically, the Scale of the Assessment and Rating of Ataxia (SARA) was used to assess the severity of clinical symptoms. The Mini-Mental State Examination (MMSE) was used to evaluate the global cognitive function. The Instrumental Activities of Daily Living (IADL) scale was employed 's to evaluate the patient’s motor and cognitive function in daily activities. Additionally, data such as age at onset, disease duration, and length of long/short segment (CAG) repeats were collected for each patient.

Patients with SCA3 were divided into 4 subgroups based on the duration of the disease. Subgroup 1 comprised asymptomatic patients with SCA3 who were identified as non-ataxic mutation carriers through positive genetic testing (SARA < 3) [16, 17]. Subgroups 2, 3, and 4 consisted of patients with disease duration from ataxia onset ≤ 5 years, 6–10 years, and ≥ 11 years, respectively (Table 1). The SCA3 patients of each subgroup were classified into four subtypes based on their clinical features [18]. For comparison, four control groups were selected from the pool of healthy controls (HC), ensuring precise matching in both age and gender with their respective patient subgroups. To maintain consistency, the sample size for each control group was set at twice that of the corresponding patient subgroup, with a consistent gender ratio. It is worth noting that there could be some overlap among the four control groups due to the constraints of a limited number of available healthy controls.

**Table 1:** Demographic and clinical data of each subgroup

	Subgroup1	Subgroup2	Subgroup3	Subgroup4	Total
*Patients*					
N	5	13	19	16	53
Age, y	25.20 ± 5.72	45.92 ± 10.68	38.79 ± 8.88	48.25 ± 10.49	42.11 ± 11.6
Gender (M:F)	3:2	8:5	10:9	13:3	34:19
Duration, y	–	3.15 ± 1.21	7.05 ± 1.08	12.56 ± 2.58	7.83 ± 4.10
CAG	67.00 ± 2.58	63.46 ± 3.97	65.36 ± 7.92	64.94 ± 3.59	64.88 ± 5.56
Type 1		4	2	2	9
Type 2		7	11	9	27
Type 3		0	3	3	6
Type 4		2	3	2	7
SARA	0.20 ± 0.44	8.46 ± 6.34	12.13 ± 7.66	14.09 ± 9.94	11.79 ± 8.31
IADL	12 ± 0	14.23 ± 6.33	20.68 ± 9.70	21.75 ± 10.19	19.29 ± 9.46
MMSE	28.80 ± 1.30	26.61 ± 4.59	27.74 ± 2.73	27.27 ± 2.87	27.42 ± 3.22
*HCs*					
N	10	26	38	32	59
Age, y	26.20 ± 2.74	45.03 ± 10.78	38.79 ± 8.65	48.7 ± 11.50	42.66 ± 12.47
Gender (M:F)	6:4	16:10	20:18	26:6	32:27

*CAG* cytosine-adenine-guanine, *SARA* scale for the assessment and rating of ataxia, *IADL* instrumental activities of daily life, *MMSE* mini-mental state examination, *HCs* healthy controls. Type 1 presents with prominent pyramidal and extrapyramidal symptoms, with mild cerebellar ataxia. Type 2 is characterized by severe cerebellar ataxia, dysarthria, and pyramidal signs. Type 3 involves cerebellar ataxia along with peripheral neuropathy, muscle atrophy, and areflexia. Type 4 is primarily defined by Parkinsonism

*There was no significant difference in age and gender between patients of SCA3 and HCs (p < 0.05). The number of HC was twice as large as the number of patients in the corresponding subgroup

### MRI data acquisition

All MRI data were collected on a Siemens Magnetom TrioTim 3.0 T MRI scanner using a 8-channel phased-array head coil. The three-dimensional structural MRI scans were acquired using a magnetization-prepared rapid acquisition gradient-echo sequence with the following parameters: repetition time (TR) = 1900 ms, echo time (TE) = 2.52 ms, inversion time = 900 ms, slice thickness = 1 mm, flip angle = 9°, matrix = 256 × 256, voxel size = 1 × 1 × 1mm^3^ and 176 slices. The diffusion tensor imaging scans were acquired using an echo planar imaging sequence with the following parameters: TR = 10,000 ms, TE = 92 ms, 64 diffusion directions (b = 1000 s/mm^2^), matrix = 128 × 128, flip angle = 90°, voxel size = 2 × 2 × 2 mm^3^ and axial slices = 65.

### Tract-based spatial statistics

Diffusion tensor imaging data were processed using the Functional Magnetic Resonance Imaging of the Brain (FMRIB) Software Library (FSL; fsl.fmrib.ox.ac.uk/fsl/). Diffusion tensor imaging scans of each participant were initially corrected for head motion and eddy currents using affine alignment, followed by brain extraction utilizing the Brain Extraction Tool. The fractional anisotropy (FA) maps of each participant were generated by applying a diffusion tensor model to each voxel and then applying intergroup voxel analysis using a TBSS program in FSL [19]. Specifically, we spatially aligned the FA map of each participant to the Montreal Neurological Institute using FMRIB's nonlinear image alignment tool. Subsequently, an average FA image was created by combining all aligned FA images and skeletonized to derive an average FA skeleton, with an FA threshold set at > 0.2. The resulting average FA skeleton was used to project the aligned FA maps of each participant, and voxel statistics were conducted on this projected data.

Voxel-wise contrasts for the FA maps were conducted for each pair of groups using a general linear model (GLM) in FSL. The GLM included group, gender, and age as covariates for each contrast. Group-wise statistics were determined using a nonparametric permutation test with 5,000 repetitions. Multiple comparison correction was applied using threshold-free cluster enhancement (TFCE), setting the significance level at corrected p < 0.05.

Using the pooled data of the four patient groups, we conducted voxel-wise correlation analyses to investigate the associations between FA and clinical parameters, including CAG, SARA, IADL, and MMSE scores. The same procedure that was used for between-group contrasts was repeated for the thresholding and reporting of the voxel-wise correlational results.

### Vertex-based shape analysis

Vertex-based shape analyses of subcortical structures, including the thalamus, hippocampus, amygdala, putamen, pallidums, caudate, nucleus accumbens and brainstem, were conducted using the FIRST procedure in FSL (http://www.fmrib.ox.ac.uk/fsl/first/index.html). Employing a deformable mesh model, FIRST generated a surface mesh for each subcortical structure, comprising vertices and triangles [20]. The number of vertices was standardized for each structure to facilitate spatial comparison across participants [20]. Despite vertex correspondence, the surface mesh, residing in the native space of each participant, might exhibit arbitrary orientation and position. To mitigate potential pose differences (rotation and translation), the surface mesh for each participant was aligned with the mean surface in standard space, achieved by minimizing the sum-of-squares difference between the participant's surface and the mean surface.

Spatial location contrasts for each vertex within each subcortical structure were conducted for each pair of groups in Montreal Neurological Institute (MNI) space utilizing a GLM in FSL. Each contrast was incorporated into a vertex-wise GLM with group, gender, and age as covariates. A nonparametric permutation test, comprising 5,000 repetitions, was subsequently employed for group-wise statistical analyses. To account for multiple comparisons, the results underwent correction for family-wise error (FWE), setting the significance threshold at corrected p < 0.05.

Using the pooled data of the four patient groups, we performed vertex-wise correlation analyses to investigate the associations between the shape of subcortical structures and clinical parameters, including CAG repeats, SARA, Instrumental Activities of Daily Living (IADL), and MMSE scores. To achieve this, a vertex-wise GLM was employed, incorporating each clinical parameter as a covariate. The results underwent correction for multiple comparisons using FWE. The significance level was set at corrected p < 0.05.

## Results

### TBSS analysis

**Fig. 1:**
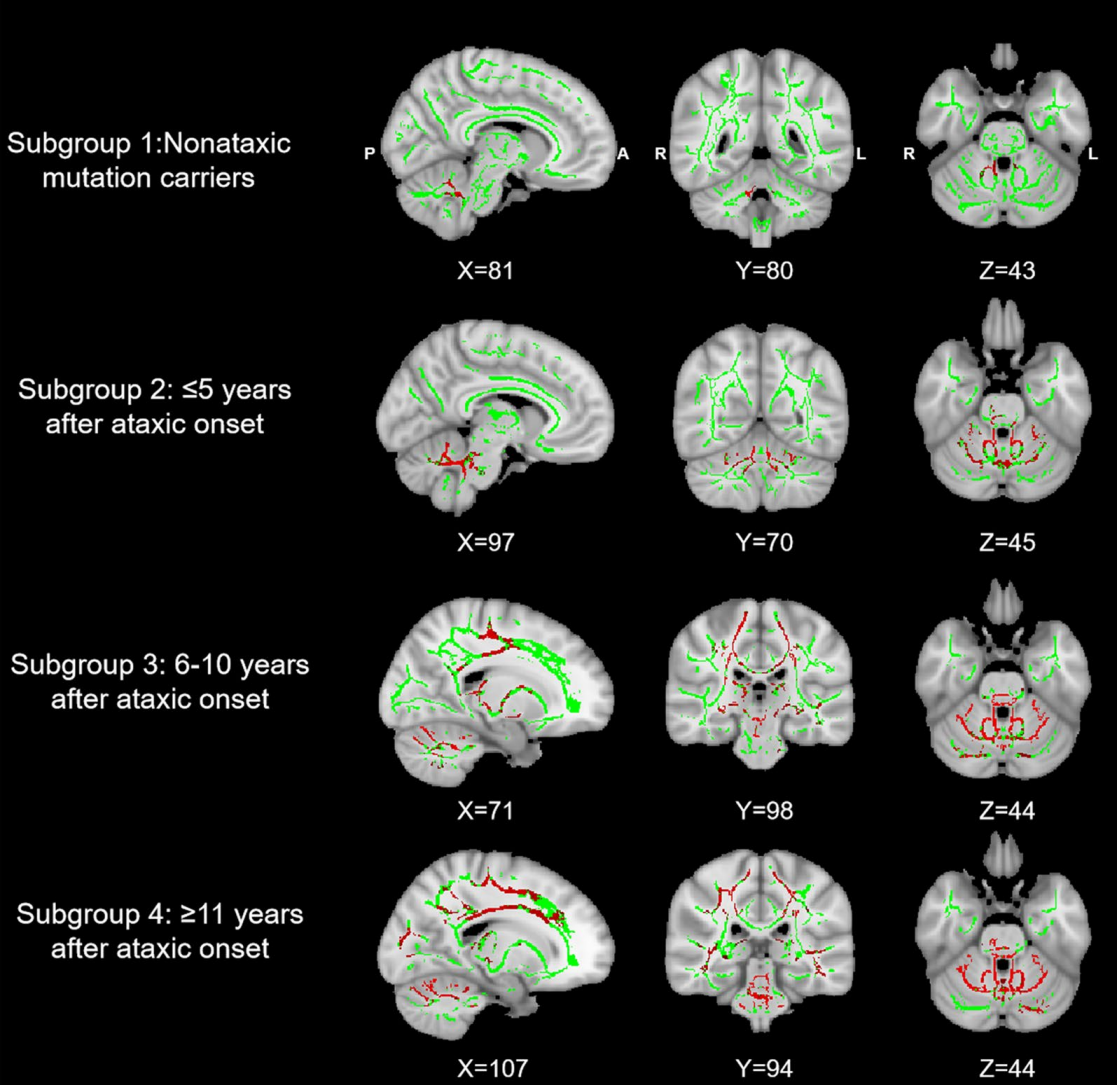
FA reductions in subgroups of SCA3 compared with their matched healthy controls. The results were TFCE-corrected

We uncovered a clear progressive pattern of white matter microstructural abnormalities along the disease course (Fig. 1). Specifically, compared with HC, asymptomatic patients (i.e., subgroup 1) showed focal FA reductions across the cerebellar peduncle (i.e., inferior, middle and superior). Patients with a disease duration of less than 5 years (subgroup 2) showed additional FA reductions in the pontine crossing tract, the corpus callosum, the left corticospinal tract, the right medial lemniscus, and the left superior corona radiata. Patients with a disease duration greater than 5 years (subgroups 3 and 4) showed more extensive FA reductions in regions typical for the preceding stages, with additional involvement in the anterior internal capsule, the posterior thalamic radiation, and the longitudinal fasciculus.

### Vertex-based shape analysis

**Fig. 2:**
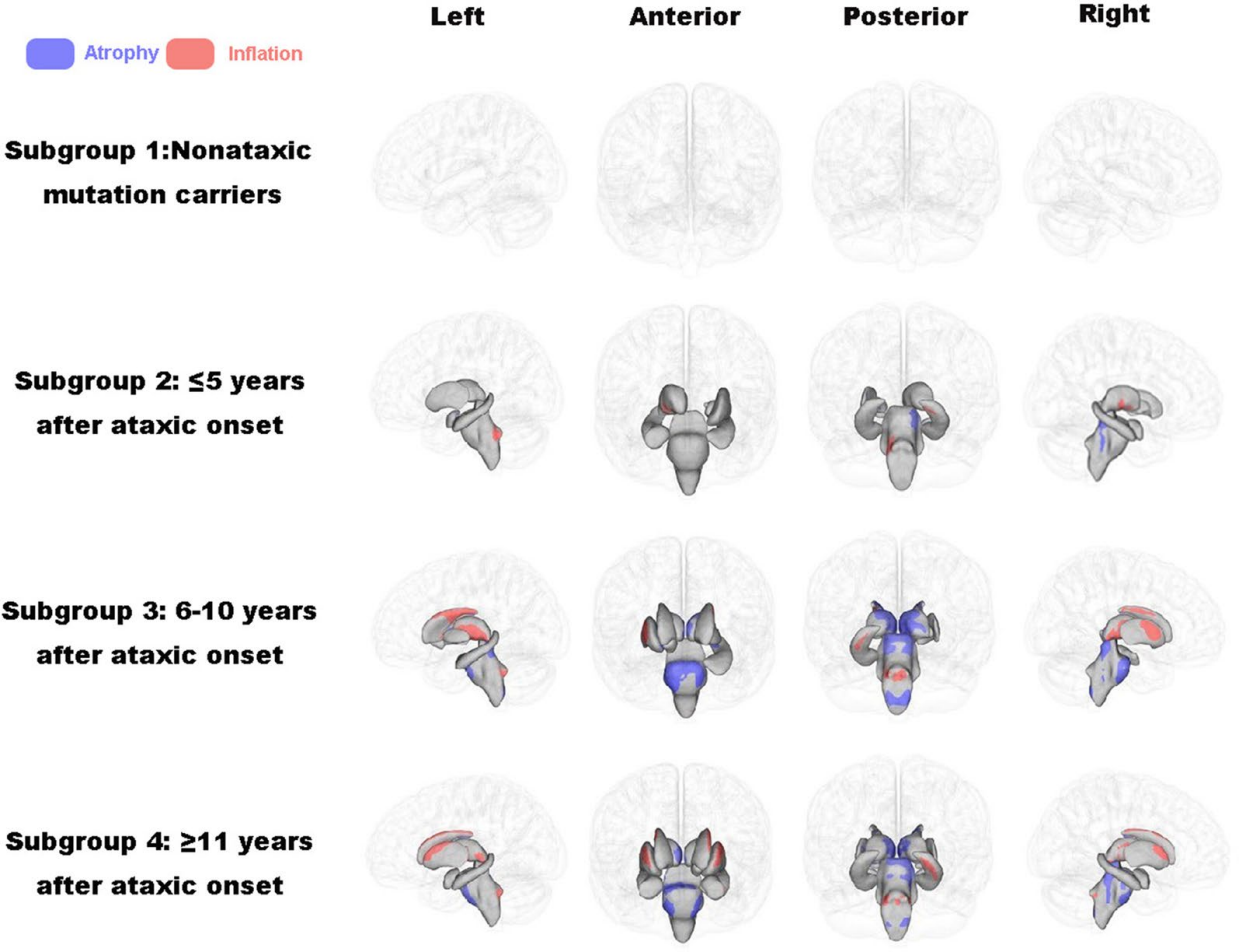
Localized subcortical shape abnormalities in subgroups of SCA3 compared with their matched healthy controls. The results were FWE-corrected

There were no significant shape differences for the subcortical structures between asymptomatic carriers (subgroup 1) and their matched healthy controls. There was an increasing number of subcortical structures affected in symptomatic patients as the disease duration increased, with concurrent inward and outward shape changes representing regional atrophy and inflation, respectively (Fig. 2). Specifically, patients with a disease duration less than five years (subgroup 2) showed significant regional atrophy in the dorsolateral brain stem, the medial part of the left pallidum and putamen, the superior caudal part of the bilateral hippocampus and right amygdala, as well as regional inflation in the inferior caudal part of the bilateral hippocampus, left dorsolateral pontine, and ventrolateral part of the right thalamus. Patients with a disease duration greater than 5 years (subgroups 3 and 4) showed more extensive shape abnormalities in regions typical for the preceding stages, with additional regional atrophy in the ventral pontine, dorsal medulla oblongata, medial part of the bilateral thalamus and bilateral putamen, medial caudal part of the bilateral caudate nucleus, and medial part of the left nucleus accumbens. Regional inflation was seen in the lateral part of the right nucleus accumbens, the lateral caudal part of the bilateral caudate nucleus, the lateral part of the bilateral thalamus and bilateral putamen, the left amygdala, and the lateral part of the bilateral pallidum.

### Correlation analyses

**Fig. 3:**
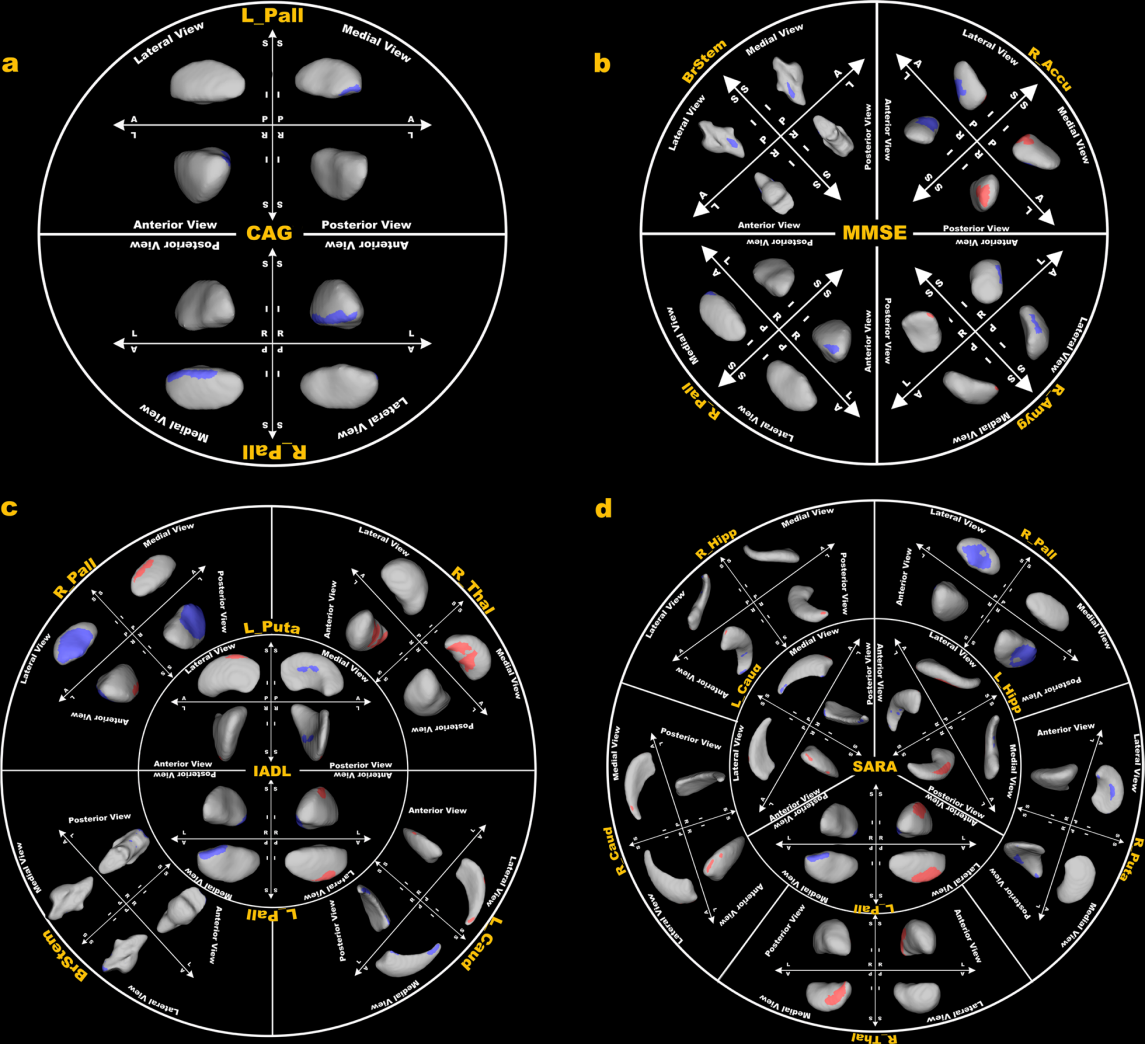
Correlations of subcortical shape with the length of CAG repeats (**a**), the MMSE score (**b**), the IADL score (**c**) and the SARA score in patients with SCA3 (**d**). Significant regions were indicated by colored areas, with red and blue respectively representing significant positive and negative correlations. The results were FWE-corrected. L, left; R, right; Pall, pallidum; BrStem, brainstem; Accu, accumbent; Amyg, amygdala; Thal, thalamus; Puta, putamen; Caud, caudate; Hipp, hippocampus

There were significant bidirectional correlations between the shape of subcortical structures and clinical data in patients with SCA3 (Fig. 3; Table S1). Specifically, the CAG score correlated negatively with the shape of the medial anterior segment of the bilateral pallidum (Fig. 3a). The MMSE score correlated negatively with the shape of the lateral segment of the brainstem, the anterior segment of the right pallidum, the lateral inferior segment of the right nucleus accumbens, the lateral segment of the right amygdala, and positively with the shape of the medial posterior segment of the right nucleus accumbens and the superior posterior segment of the right amygdala (Fig. 3b). The IADL score correlated negatively with the shape of the superior posterior segment of the brainstem, the medial posterior segment of the left caudate, the lateral segment of the right pallidum and the medial segment of the left pallidum and putamen, and positively with the shape of the medial segment of the right thalamus, the medial segment of the right pallidum, the lateral anterior segment of the left caudate and the lateral segment of the left pallidum and putamen (Fig. 3c). The SARA score correlated negatively with the shape of the lateral segment of the right pallidum and putamen, the anterior segment of the right hippocampus, the medial posterior segment of the left caudate, the medial anterior segment of the left hippocampus and the medial segment of the left pallidum, and positively with the shape of the medial anterior segment of the right caudate, the medial segment of the right thalamus, the posterior segment of the right hippocampus, the anterior segment of the left caudate, the posterior segment of the left hippocampus and the lateral segment of the left pallidum (Fig. 3d). There was no significant correlation between between FA and clinical scores.

## Discussion

In this study, we characterized the progressive pattern of localized shape abnormalities in subcortical structures in SCA3 as white matter microstructural abnormalities expanded from initial focal changes in the cerebellum to encompass broader regions throughout the brain. The main findings are summarized as follows. First, TBSS analyses revealed a progressive pattern of white matter microstructural abnormalities throughout the course of SCA3 as indicated by an expanding topographic distribution of FA reductions (originating from the cerebellar peduncle) in patients with a longer duration of illness. Second, we revealed a consistent progressive pattern of subcortical shape abnormalities throughout the course of SCA3, as evidenced by a greater number of structures and a more extensive distribution of regions exhibiting significant bidirectional shape changes in symptomatic patients with longer duration of illness. There were no significant shape changes in asymptomatic patients. Last, we uncovered significant bidirectional correlations between subcortical localized shape changes and clinical variables in patients with SCA3, including CAG, SARA, MMSE and IADL scores.

In the present study, we found significantly reduced FA in the cerebellar peduncle of individuals in the asymptomatic phase of SCA3. The result is in good agreement with previous studies, which showed significant reductions in FA, and gray/white matter volume [21–24]. Our finding likely suggests that microstructural abnormalities in the white matter of cerebellar peduncle precedes the onset of clinical motor symptoms in SCA3, with the cerebellar peduncle a potential focal onset point of this disorder. Furthermore, this study uncovered a clear progressive pattern of white matter microstructural abnormalities in symptomatic patients with SCA3, in keeping with previous findings of a significant negative correlation between FA and disease duration [25, 26]. These findings suggest that FA may serve as a potential biomarker for monitoring the progression of SCA3. As no significant correlation was found between FA and the clinical measurements in this study, the specific role of the white matter microstructural abnormalities in contributing to the progression of clinical symptoms in these patients remains unknown.

Using vertex-based analysis, the present study uncovered a progressive pattern of subcortical shape abnormalities in patients with SCA3 as white matter microstructural changes extended from a focal abnormality in the cerebellar peduncle to encompass more widely-distributed fiber tracts. To the best of our knowledge, this study is the first to examine the localized shape abnormalities of subcortical structures in patients with SCA3. Unlike prior volumetric studies that reported mixed findings of unidirectional subcortical atrophy or no volumetric changes [23, 27], we found significant bidirectional shape changes in symptomatic patients in a number of subcortical structures, including the bilateral thalamus, caudate, putamen, pallidum, hippocampus and the brainstem, representing a coexistence of both outward inflation and inward atrophy. The mechanisms underlying such inconsistency remain unknown and may relate to sample heterogeneities (such as ataxia, pyramidal, extrapyramidal and Parkinsonism symptoms) among studies or a high sensitivity of surface-based vertex-wise shape analysis relative to whole-brain voxel-based morphometry or ROI-based volumetric analyses [28]. Given that Type 2 was the most common subtype across the three symptomatic groups, the observed subcortical shape changes may primarily reflect the pathological characteristics of the pathological features typical of this subtype. The coexistence of both inward atrophy and outward inflation likely indicates the occurrence of both neuropathologic and compensatory processes [29] in relevant subcortical structures, with the regional outward inflation potentially reflecting adaptive changes to compensate for the white matter microstructural abnormalities and/or regional atrophy existing in the same or other subcortical structures. As the disease progresses, the compensatory processes may deplete eventually [30], leading to the emergence of universal, unidirectional atrophy in more advanced stages. In contrast to a significant FA reduction in the asymptomatic phase, we found no significant shape changes in the subcortical structures in asymptomatic individuals, which likely demonstrates a sequential order of these abnormalities [25], with the subcortical shape changes being secondary to the progression of white matter microstructural abnormalities. Overall, the present study uncovered significant localized bidirectional shape changes in the subcortical structures of patients with SCA3 across different disease stages. These findings provided some new insights into the critical involvement of subcortical structures in the pathophysiology of this disorder.

Using the pooled data of the four patient subgroups, we found significant bidirectional correlations between localized shapes of subcortical structures and clinical data. Specifically, the CAG score indexing the length of CAG amplification was negatively correlated with the shape of the medial anterior segment of the bilateral pallidum, suggesting a longer length of CAG amplification with greater inward atrophy of this region in patients. The neural mechanisms underlying this relationship remain unknown. It is likely that an increased number of CAG repeats in the *ATXN3* gene results in a longer polyglutamine tract in the ataxin-3 protein, facilitating the formation of neurotoxic protein aggregates and, in turn, contributing to neuronal death within the pallidum in patients with SCA3 [2]. The possible relationship between polyglutamine tract length and neurotoxic protein aggregates merits further study. This study also found significant bidirectional correlations between SARA score and the shape of caudate, pallidum, putamen, and thalamus, in keeping with the bidirectional shape changes observed in the four patient subgroups. Indeed, the caudate, pallidum, and putamen are key components of the basal ganglia and play important roles in regulating voluntary movements and maintaining motor coordination [31]. The thalamus, a major relay station for sensory and motor signals, plays crucial roles in relaying basal ganglia output to the cortical motor fields, providing essential input for the initiation and modulation of voluntary movements [32]. Our finding of bidirectional correlations between SARA score and the shape of caudate, pallidum, putamen and thalamus suggests a role of these subcortical structures in contributing to the complex and heterogeneous motor phenotype in SCA3. Moreover, we identified significant bidirectional correlations between the IADL score and the shape of caudate, pallidum, putamen, and thalamus, akin to the correlations observed between the SARA score and the shape of these subcortical structures. Given that the IADL score is an integrated measurement of individuals' motor skills and cognitive functioning in managing the demands of daily life [33, 34], such correlations likely indicate that these subcortical structures are not only fundamental to motor functions but also play key roles in cognitive functions. This speculation is partially supported by the finding of bidirectional correlations between MMSE score and the shape of pallidum and putamen in these patients, although the significant regions identified in the two correlation analyses did not overlap. Taken together, these correlational findings, along with the between-group differences, suggest that the localized shape changes in subcortical structures may have contributed to the complex and heterogeneous motor and nonmotor phenotypes in patients with SCA3.

This study had several limitations. First, the sample size of the patient cohort in this study was small, albeit comparable with previous investigations. Consequently, the validation of our results in larger samples is imperative. Second, the cross-sectional design of our study limits the exploration of the dynamic changes in white matter microstructure and shape of subcortical structures in SCA3. Future longitudinal studies are needed to further delve into these problems. Thirdly, due to the lack of appropriate tools for analyzing cerebellar shape, this study was unable to examine localized shape abnormalities in the cerebellum of these patients. Given the critical role of cerebellar abnormalities in the pathophysiology of this disorder, future research should focus on developing new analytical methods for cerebellar shape and investigating local cerebellar shape abnormalities in these patient subgroups [35, 36].

In conclusion, we demonstrated progressive, localized, and bidirectional changes in the shape of subcortical structures that related to diverse clinical manifestations in SCA3. This study suggests the critical involvement of localized subcortical shape changes in contributing to the clinical heterogeneity of SCA3 and has potential implications for the development of novel therapeutic interventions targeting specific subcortical nuclei in this disorder.

## Supplementary Information

**Additional file 1.**

## Data Availability

The data sets used or analyzed during the current study are available from the corresponding author on reasonable request.
