# Supplementary material for: Progressive subcortical involvement as spinocerebellar ataxia type 3 advances
Source: Orphanet J Rare Dis. 2025 Jun 4;20:275. doi: 10.1186/s13023-025-03803-3 (PMC12135544; doi:10.1186/s13023-025-03803-3)
Supplement: Supplementary file 1 — Additional file 1. [file 13023_2025_3803_MOESM1_ESM.docx]

Table S1. Associations between subcortical shape and clinical scores

| **Clinical Score** | **Structure** | **Hemisphere** | **Direction of Correlation** | **Anatomic Location** |
| --- | --- | --- | --- | --- |
| The length of CAG | Pallidum | Left | Negative | Medial anterior segment |
|  |  | Right | Negative | Medial anterior segment |
| MMSE | Brainstem | - | Negative | Lateral segment |
|  | Pallidum | Right | Negative | Anterior segment |
|  | Nucleus Accumbens | Right | Negative | Lateral inferior segment |
|  |  |  | Positive | Medial posterior segment |
|  | Amygdala | Right | Negative | Lateral segment |
|  |  |  | Positive | Superior posterior segment |
| IADL | Brainstem | - | Negative | Superior posterior segment |
|  | Caudate | Left | Negative | Medial posterior segment |
|  |  |  | Positive | Lateral anterior segment |
|  | Pallidum | Left | Negative | Medial segment |
|  |  |  | Positive | Lateral segment |
|  |  | Right | Negative | Lateral segment |
|  |  |  | Positive | Medial segment |
|  | Putamen | Left | Negative | Medial segment |
|  |  |  | Positive | Lateral segment |
|  | Thalamus | Right | Positive | Medial segment |
| SARA | Pallidum | Left | Negative | Medial segment |
|  |  |  | Positive | Lateral segment |
|  |  | Right | Negative | Lateral segment |
|  | Putamen | Right | Negative | Lateral segment |
|  | Hippocampus | Left | Negative | Medial anterior segment |
|  |  |  | Positive | Posterior segment |
|  |  | Right | Negative | Anterior segment |
|  |  |  | Positive | Posterior segment |
|  | Caudate | Left | Negative | Medial posterior segment |
|  |  |  | Positive | Anterior segment |
|  |  | Right | Positive | Medial anterior segment |
|  | Thalamus | Right | Positive | Medial segment |

Note: All the significant clusters were obtained with a threshold of FWE-corrected p < 0.05.
